# Supplementary material for: Impact of Physical Activity on Disability Risk in Elderly Patients Hospitalized for Mild Acute Diverticulitis and Diverticular Bleeding Undergone Conservative Management
Source: Medicina (Kaunas). 2021 Apr 8;57(4):360. doi: 10.3390/medicina57040360 (PMC8068129; doi:10.3390/medicina57040360)
Supplement: Supplementary file 1 [file medicina-57-00360-s001.zip › Table S1-STROBE table.pdf]

The Strengthening the Reporting Observational studies in Epidemiology – Molecular Epidemiology (STROBE-ME) Reporting Recommendations: Extended from STROBE statement		
**Item**	**Item number**	**STROBE Guidelines**
**Title and abstract**	1	Impact of physical activity on disability risk in elderly patients hospitalized for diverticular bleeding and mild acute diverticulitis undergone conservative management
**Introduction**		
Background rationale	2	The role of physical activity (PA) in elderly patients admitted to surgical units for mild acute diverticulitis in the development of disability has not been clarified so far. Our aim is to demonstrate the relationship between physical activity and better post-discharge outcomes on disability in elderly population.
Objectives	3	Based on the association between a normal to high exercise ability and clinically relevant benefit in physical activity after hospitalization in surgical units, we decided to use the PASE scale in order to investigate, before and after hospitalization, 1) the association of PA with post-hospital stay disability using the activity of daily living (ADL) and the instrumental activity of daily living (IADL) scale, 2) the association of PA with the risk of post-hospital stay immobilization using the Exton-Smith scale
**Methods**		
Study design	4	1). Data were prospectively collected from October 2018 to March 2020. Inclusion criteria were: 1) age of 65

		years and older 2) good daily living and instrumental activities daily living independence, as evaluated by an ADL and IADL score 3) low development risk of pressure injuries evaluated by an Exton-Smith Scale (more than 15 points), 4) patients who underwent conservative procedures.	
Setting	5	Retrospective data analysis of elderly patients admitted to General Surgery Unit of "Antonio Cardarelli" hospital, Campobasso, Italy,<br>Data were prospectively collected from October 2018 to March 2020.<br>Patients underwent hospitalization for low and mild acute diverticulitis and diverticular bleeding.	
Participants	6	Inclusion criteria were: 1) age of at least 65 years 2) good daily living and instrumental activities daily living independence, as evaluated by an ADL and IADL score 3) low development risk of pressure injuries evaluated by an Exton-Smith Scale (more than 15 points), 4) patients who underwent conservative procedures.	
Variables	7	Our analysis was corrected for age, gender and BMI.	
Data source/measurement	8	P values < 0.05 were considered statistically significant. Analysis was performed using the STATA 11.2 software (Stata Corp. LP Collage Station, Texas USA).	
Bias	9	We excluded from the study all patients who were addressed to critical care or emergency surgery due to the impossibility to complete the geriatric assessment during pre-screening,<br>All patients underwent undergone any surgical, endoscopic or radiologic	

		intervention were excluded, because surgical stress should be a potential bias.<br>We excluded patients affected by baseline delirium, physician-diagnosed dementia, Mini-Mental State Examination (MMSE) score below 24 points. Patients affected by severe auditory or visual deficits were also excluded.	
Study size	10	This is a retrospective single center study that included 56 elderly patients (over 65 years old) affected by low and mild acute diverticulitis and diverticular bleeding.	
Quantitative variables	11	Continue variables were expressed as mean ± standard deviation (SD), while categorical variables are expressed as number and percentage	
Statistical methods	12	Continue variables and categorical variables are compared using the χ2 test.	
		The Bonferroni ANOVA test with post-hoc analysis was used for multiple comparisons.<br>Logistic regression analysis was performed to evaluate the association between PASE and post-hospitalization disability development.	
**Results**			
Participants	13	We enrolled 56 elderly patients. Mean age was of 75.9 ± 8.91 years old; 57.1% were males. Population baseline characteristic are showed in table 1.	
Descriptive data	14	Mean age was of 75.9 ± 8.91 years old; 57.1% were males. Population baseline characteristic are showed in table 1.	

Outcome data	15	Population baseline characteristics were divided in three groups as presented in Table 3.	
Main results	16	BMI had a good impact on physical activity. Our results showed that an higher BMI is associated with more physical active patients (p=0.044). A better renal function follow the same trend. On the other hand, we observed a loss of activity in patients who had previous underwent oncologic surgery.<br>Regarding geriatric evaluations we compared pressure injury development and immobilization to ADL, IADL and Exton-Smith Scale. We consider cognitive performance reduction with a cut off of loss of more than 5 points in the Short Port of ADL and IADL and a loss of more than 15 points showed in the Exton-Smith Scale,(p-value 0.017 and 0.010, respectively).	
**Discussion**			
Key results	18	We observed a significant association between pre-admission physical activity and post-discharge reduction of cognitive performance.	
Limitations	19	This is an observational study, single-centred with a limited number of participants and our data should be confirmed in future larger studies.	
Interpretation	20	Reduced physical activity in every day life, as indicated by PASE score, in elderly patients is associated with increased post-recovery disability regarding independence, cognitive performance and immobilization.	
Generalisability	21	The physical dysfunction should be carefully managed among multidisciplinary teams to achieve better outcomes in elderly patient	
