# Supplementary figures and images for: Impact of Physical Activity on Disability Risk in Elderly Patients Hospitalized for Mild Acute Diverticulitis and Diverticular Bleeding Undergone Conservative Management
Source: Medicina (Kaunas). 2021 Apr 8;57(4):360. doi: 10.3390/medicina57040360 (PMC8068129; doi:10.3390/medicina57040360)

## FLUX DIAGRAM STROBE

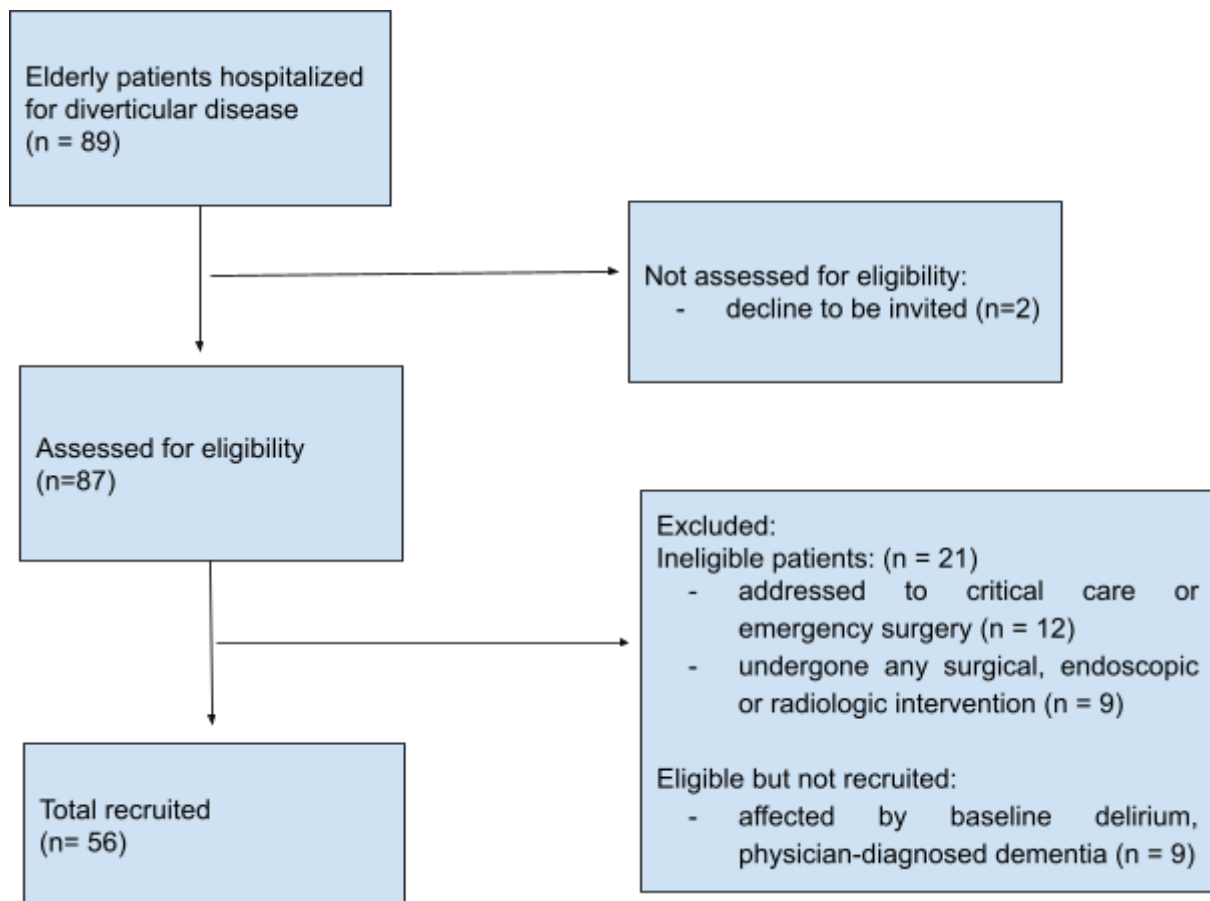

Supplement: Supplementary file 1 [file medicina-57-00360-s001.zip › Figure S1-FLUX DIAGRAM STROBE.pdf]
