# Supplementary material for: Impact of Physical Activity on Disability Risk in Elderly Patients Hospitalized for Mild Acute Diverticulitis and Diverticular Bleeding Undergone Conservative Management
Source: Medicina (Kaunas). 2021 Apr 8;57(4):360. doi: 10.3390/medicina57040360 (PMC8068129; doi:10.3390/medicina57040360)
Supplement: Supplementary file 1 [file medicina-57-00360-s001.zip › Scheda Paziente File S1.doc]

**SCHEDA PAZIENTE**

**DATA ARRUOLAMENTO ___/___/______**

**NOME: ___________________________ COGNOME: _________________________________
DATA DI NASCITA: ___/___/________ RECAPITI: __________________________________**

ANAGRAFICA

| **Numero cartella clinica** |  | **Codice Paziente** |  |
| --- | --- | --- | --- |
| **Codice Fiscale** |  | **Età** |  |
| **Altezza** |  | **Peso** |  |
| **CIRCUM. Vita** |  |  |  |

| **Pa sist/Pa diastolica** | **/** | **Frequenza cardiaca** |  |
| --- | --- | --- | --- |
| **Glasgow Coma Scale** |  | **Killip Class** |  |
| **NYHA** |  |  |  |

ANAMNESI PATOLOGICA PROSSIMA

**________________________________________________________________________________________________________________________________________________________________________________________________________________________________________________________________________________________________________________________________________________________________________________________________________________________________________________________________________________________________________________________________________________________________________________________________________________________________________________________________**

ANAMNESI PATOLOGICA REMOTA

**TERAPIA**

| **TERAPIA in DOMICILLIO** |  | **TERAPIA attuale** |  |
| --- | --- | --- | --- |
| **FARMACO** | **DOSE** | **FARMACO** | **DOSE** |
|  |  |  |  |
|  |  |  |  |
|  |  |  |  |
|  |  |  |  |
|  |  |  |  |
|  |  |  |  |
|  |  |  |  |
|  |  |  |  |
|  |  |  |  |
|  |  |  |  |

**PARAMETRI DI LABORATORIO** data (__/__/____)

| **Emoglobina** |  | **Eritrociti** |  |
| --- | --- | --- | --- |
| **Leucociti** |  | **Glicemia** |  |
| **Creatinina** |  | **BUN(Urea/2,14)** |  |
| **AST** |  | **Urea** |  |
| **ALT** |  | **AMS** |  |
| **Fibrinogeno** |  | **VES** |  |
| **PCR** |  | **Colinesterasi** |  |
| **Colesterolo totale** |  | **Col. LDL** |  |
| **Col. HDL** |  | **Trigliceridi** |  |
| **Acido Urico** |  | **Bilirubina totale** |  |
| **ALBUMINA** |  | **Na** |  |
| **Proteine totali** |  | **K** |  |
| **GFR (epi)** |  | **Ca** |  |
| **procalcitonina** |  | **Bilirubina totale** |  |
| **ferritina** |  |  |  |
| **folati** |  |  |  |

**Scala PASE**

| **ATTIVITA’** | **QUANTITA’** | **PESO** |
| --- | --- | --- |
| **Camminare e/o bicicletta** | **ore/giorno** | **20** |
| **Attività sportive e/o ricreative**   - **Leggere** - **Moderate** - **Pesanti** |  |  |
|  | **ore/giorno** | **21** |
|  | **ore/giorno** | **23** |
|  | **ore/giorno** | **23** |
| **Esercizi in palestra** | **ore/giorno** | **30** |
| **Lavoro saltuario** | **si/no** | **21** |
| **Lavoro casalingo leggero** | **si/no** | **25** |
| **Lavoro casalingo pesante** | **si/no** | **25** |
| **Riparazioni in casa** | **si/no** | **30** |
| **Lavoro nei campi** | **si/no** | **36** |
| **Giardinaggio** | **si/no** | **20** |
| **Caregiver** | **si/no** | **35** |

| **ATTIVITÀ FISICA** | **PUNTEGGIO PASE** |
| --- | --- |
| INATTIVITÀ | <42 |
| SCARSA | 43-105 |
| MODERATA | 106-145 |
| FISICA INTENSA | >146 |

**MPI Multidimensional Prognostic index**

| **Ulteriori dati MPI** |  |
| --- | --- |
| Numero farmaci: |  |
| Con chi vive: | in famiglia |
|  | da solo |
|  | In residenza |

GERIATRIC DEPRESSION SCALE (GDS)

| **Valutazione Affettiva** | 1 | 0 |
| --- | --- | --- |
| E’ fondamentalmente soddisfatto della sua vita? | NO | SI |
| Ha abbandonato molte delle sue attività o interessi? | SI | NO |
| Sente che la sua vita è vuota? (Ha l’impressione che…) | SI | NO |
| Si sente spesso annoiato? | SI | NO |
| E’ di buon umore per la maggior parte del tempo? | NO | SI |
| Teme che possa succederle qualche cosa di male ? (di brutto?) | SI | NO |
| Si ritiene nel complesso felice (contento?) | NO | SI |
| Si sente spesso non aiutato (senza aiuto, senza appoggio?) | SI | NO |
| Preferisce stare in casa piuttosto che uscire a fare cose nuove (diverse dal solito?) | SI | NO |
| Pensa di avere più problemi di memoria degli altri (suoi coetanei?) | SI | NO |
| Pensa che sia bello stare al mondo (vivere) in questo momento ? | NO | SI |
| Pensa che attualmente la vita che fa abbia poco senso (sia quasi senza valore?) | SI | NO |
| Si sente pieno (carico) di energia? | NO | SI |
| Ritiene che la sua situazione (condizione) sia senza speranza ? | SI | NO |
| Pensa che gli altri siano meglio di lei (in condizioni migliori?) | SI | NO |
| Punteggio: 0-3 punti (normale) 4-7 punti (depressione lieve) 8-11 punti (depressione moderata) 12-15 punti (depressione clinicamente rilevante) | | |
